# Neutrophil-to-Lymphocyte Ratio and Platelet-to-Lymphocyte Ratio: Novel Markers for Diagnosis and Prognosis in Patients with Idiopathic Sudden Sensorineural Hearing Loss

**DOI:** 10.1155/2014/702807

**Published:** 2014-05-07

**Authors:** Young Joon Seo, Jun hui Jeong, Jae Young Choi, In Seok Moon

**Affiliations:** Department of Otorhinolaryngology, Yonsei University College of Medicine, 250 Seongsanno, 134 Shinchon-dong, Seodaemun-gu, Seoul 120-752, Republic of Korea

## Abstract

*Background.* We aim to provide useful evidence about the association of neutrophil-to-lymphocyte ratio (NLR) and platelet-to-lymphocyte ratio (PLR) with idiopathic sudden sensorineural hearing loss (ISSNHL) and its possibility of emerging as a cheap, reliable, and independent prognostic marker of ISSNHL. * Methods.* 348 patients diagnosed with ISSNHL were included in our retrospective data analysis. Blood samples and the hearing assessments of the patients were carried out. Then, the patients were divided into 2 groups as “recovered” and “unrecovered” according to their response to the treatment. * Results.* Both mean NLR and PLR values of the ISSNHL patients were significantly higher than the control group (both *P* < 0.001). The NLR value was 5.98 ± 4.22 in the unrecovered group and 3.50 ± 3.38 in the recovered group (*P* < 0.001). After adjustment in a binary logistic regression model, only NLR value was associated with the recovery of ISSNHL (*P* = 0.001). * Discussion.* We demonstrated for the first time that NLR and PLR values were significantly high in ISSNHL patients. Also the NLR level might be taken into account as a novel potential marker to predict the patients' prognosis in terms of recovery.

## 1. Introduction

A common definition of idiopathic sudden sensorineural hearing loss (ISSNHL) is a loss of greater than 30 dB in 3 contiguous frequencies in less than 3 days [1]. A number of etiologies of ISSNHL have been proposed, including viral infection, vascular disturbance, and immune-mediated mechanisms. However, there is no conclusive evidence for any particular hypothesis [2–4]. The cause of ISSNHL has been focused on chronic inflammation recently [5]. Chronic inflammation can cause microvascular injury and atherogenesis, which is increasing ischemic risk in a direct manner [6]. The recent remarkable observation has been that neutrophil-to-lymphocyte ratio (NLR) has predictability as a marker in cardiovascular diseases and is emerging as an independent useful prognostic parameter in cardiovascular diseases [7]. And platelet-to-lymphocyte ratio (PLR) is associated with poor prognosis in patients with a key role in atherosclerosis and atherothrombosis in peripheral arterial occlusive disease patients [8, 9]. Both increasing NLR and PLR values may be risk factors for atherosclerosis as well as inflammation in microvascular structures.

In this study, we aimed to investigate the association of NLR and PLR with ISSNHL and to provide its usability of emerging as a cheap, reliable, and independent prognostic marker of ISSNHL, for the first time.

## 2. Materials and Methods

### 2.1. Subjects

We reviewed 1046 consecutive ISSNHL patients treated in our department from 2009 to 2013 in our retrospective data analysis. We defined ISSNHL as an acute unilateral deafness, with an abrupt onset (generally within 3 days), of more than 30 dB hearing loss at three consecutive frequencies [10]. Inclusion criteria for this study were as follows: visiting within 1 week from onset, no previous steroid treatment, taking blood samples and pure tone hearing tests at the first visit, and no abnormal findings causing hearing loss on the MRI. Patients were excluded from the study if they had any acute inflammation; infection; diabetes mellitus; systemic hypertension; hyperlipidemia; coronary artery disease; acute or chronic renal failure; chronic liver disease; chronic obstructive pulmonary disease; connective tissue disease; inflammatory bowel disease; and any otologic disease such as chronic otitis media, otosclerosis, acoustic trauma history, and Meniere's disease. 348 of 1046 were finally enrolled in this study. All the patients were treated with prednisone in the dose of 1 mg/kg per day, with a progressive dose reduction, maintained for at least 2 weeks with dexamethasone injections intratympanically. To compare the biomarker profile, 537 age- and sex-matched healthy people with no hearing loss who came to our hospital for regular health checkup were also included in the present study. All of them have no acute inflammations or otologic diseases of above criteria. The study was approved by the International Review Board (IRB) of the Medical University of Yeonsei (IRB number 4-2013-0935).

### 2.2. Hematologic Examinations

Blood samples were tested for all patients at the first visit to prevent adverse effects of the steroid treatment in high risk patients like patients with DM, CBC, and routine chemistry. NLR, PLR were calculated as a simple ratio between the absolute neutrophil and the absolute lymphocyte counts, the absolute platelet and the absolute lymphocyte counts. An automated blood cell counter was used for CBC measurements using Sysmex XE-2100 (Sysmex, Kobe, Japan). All samples were run in duplicate, and the mean values were used for statistical analysis.

### 2.3. Auditory Evaluation

Pure tone thresholds were obtained for air conduction at 250 Hz, 500 Hz, 1 kHz, 2 kHz, 4 kHz, and 8 kHz and for bone conduction at 250 Hz, 500 Hz, 1 kHz, 2 kHz, and 4 kHz, respectively. Audiologic data were reported by the recommended methods of the Hearing Committee of the American Academy of Otolaryngology Head and Neck Surgery. Patients were evaluated according to the recovery that was seen in a maximum of about two months in the follow-up period. PTA was done with responses of the patients to the treatment being classified according to Siegel's [11, 12] criteria (Table 1). Then, the patients were divided into 2 groups as “recovered (complete + partial + slight)” and “unrecovered (no improvement)”.

### 2.4. Statistical Analyses

Power analysis was performed to determine the extent to which the proposed sample size would be adequate (effect size = 0.083, *α*-risk = 0.05, and sample size = 357). Continuous variables were summarized as mean ± standard deviation (SD) and comparisons between continuous variables utilized the Student's *t*-test. Categorical variables were summarized as percentages of the group total and comparisons between groups were analyzed using either Fisher exact test or chi-square test where appropriate. Assessment of the bivariate relationship between recovery and each risk factor was performed using data from 348 patients. We further calculated odds ratios (OR) with 95% confidence intervals (CI) for unrecovered ISSNHL-risk factors with a binary logistic regression model. This adjusted for confounders and enabled determination of variables of interest associated with increased risk of hearing disturbance. All tests used a *P* value of 0.05 as a threshold for significance. All statistical analyses were performed using SPSS software (PASW for Windows, Rel. 18.0.0. 2009; SPSS Inc., Chicago, IL, USA).

## 3. Results

A total of 348 ISSNHL patients were included in the current analysis. All the characteristics and laboratory data between ISSNHL patients group and control group are outlined in Table 2. Groups were similar in terms of age, sex, and body mass index (BMI) (*P* > 0.05). Mean NLR was 4.48 ± 3.92 and mean PLR was 169.25 ± 102.88 in the patients group. Both mean NLR and PLR values of the patients were significantly higher than the control group (both *P* < 0.001). Though glucose was elevated in the patients group (*P* < 0.001), which was the result of random glucose test, the values in both groups were under normal value.

ISSNHL patients were divided into two subgroups according to whether their PTAs in the follow-up periods would be recovered or not. When the recovered group was compared with the unrecovered group, age, sex, BMI, WBC, absolute neutrophil counts, absolute monocyte counts, platelet counts, BUN, Cr, and AST values were not significantly different between two groups (Table 3). Mean lymphocyte value was higher in the unrecovered group than in recovered group (*P* = 0.014). The NLR value was 5.98 ± 4.22 in the unrecovered group and 3.50 ± 3.38 in the recovered group (*P* < 0.001). The PLR value was 200.88 ± 112.12 and 148.47 ± 90.77 (*P* < 0.001). Glucose value was also significantly different in two groups (*P* < 0.001). Significant values were used as variables in a binary logistic regression model to evaluate these values as independent risk factors for recovery of ISSNHL (Table 4). In this model lymphocyte, monocyte, NLR, PLR, and glucose were additionally included. After adjustment for those values, only NLR value was associated with the recovery of ISSNHL (*P* = 0.001).

**Figure 1:**
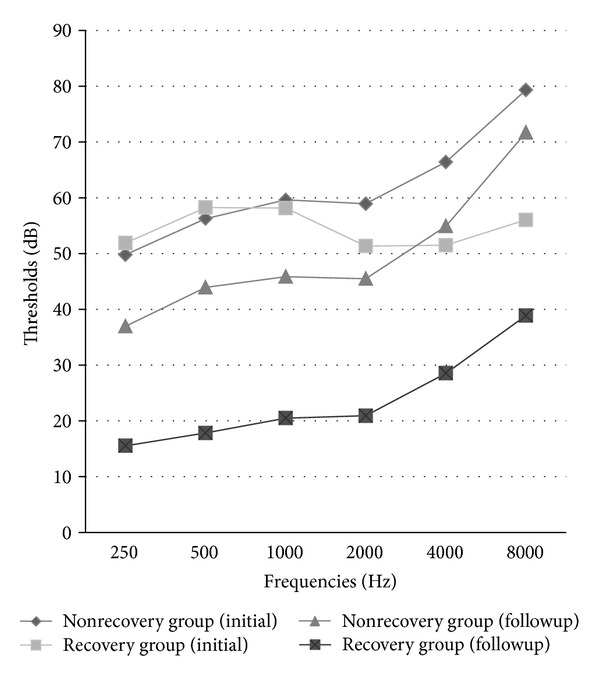
Audiometric evaluations of the recovered group and the unrecovered group at different frequencies.

In initial PTAs when they took a pure tone analysis at a first visit, we could see more high tone loss PTAs in the unrecovered group than in the recovered group (Table 5). In the unrecovered group, three frequencies of 2000, 4000, and 8000 Hz were significantly decreased, compared with the recovered group, respectively (*P* = 0.038, *P* < 0.001, and *P* < 0.001). Although there were significant changes for PTAs in all frequencies (all *P* < 0.001), low tone (250, 500 Hz) and middle tone (1000, 2000 Hz) showed remarkable recoveries at the follow-up PTAs in the recovered group (Figure 1).

## 4. Discussion

The major finding of this study is that NLR and PLR values were significantly higher in patients with ISSNHL than the control group and might be involved as the values of atherosclerosis in the pathogenesis of ISSNHL. The ISSNHL is a common otologic emergency, presenting mostly as an acute unilateral deafness with an abrupt onset and defined as sensorineural hearing loss of at least 30 dB occurring in at least 3 consecutive frequencies within 72 hours [1]. However, the exact etiopathogenesis of the ISSNHL is still unclear. Microcirculatory failure, infectious, immunologic, and inflammatory reasons have been hypothesized [3, 4]. According to the viral theory, ISSNHL is due to a herpes simplex virus infection; however, no specific serological profiles or response to antiviral treatment has been reported [2]. An immune-mediated hypothesis also cannot explain why impaired immunity and autoimmune causes are not observed in every case [13].

Interestingly, ISSNHL could also be the result of an ear microcirculation alteration due to genetic prothrombotic susceptibility or cardiovascular risk factors such as hypertension and diabetes [14, 15]. A higher blood viscosity may determine damage in ear microcirculation that can produce hearing loss [16, 17]. Ciccone et al. showed that ISSHL seemed to be associated with vascular endothelial dysfunction and preclinical atherosclerosis by calculating the carotid intima-media thickness [18]. Capaccio et al. studies examined hypercoagulable genomic polymorphisms and found statistically significant differences in patients suffering from ISSNHL with and without documented cardiovascular thrombotic disease [19]. WBC and its subtypes were found as inflammatory markers in cardiovascular diseases [20]. NLR was defined as a novel potential marker to determine inflammation in cardiac and noncardiac disorders; oncology; cardiology (acute coronary syndromes, heart failure, and coronary revascularization procedures); end-stage renal disease; and inflammatory diseases such as Alzheimer, ulcerative colitis, and appendicitis [21, 22]. A higher NLR indicates a higher level of inflammation [23]. The interrelation between neutrophils and endothelium has been accused of causing increased damage to the endothelium and reported by Ott et al. to explain platelet adhesion in patients with unstable angina [24].

Platelets play an important role in the progression of atherosclerosis. An elevated platelet count leading to an elevated PLR might therefore lead to an increase in vascular endpoints. Gary et al. proved that an increased PLR is significantly associated with patients at high risk for critical limb ischemia (CLI) and other cardiovascular endpoints [8]. By means of NLR and PLR CLI patients with a high risk for limb amputation during a five-year follow-up period were discriminated from patients with a lower risk for limb amputation during the same follow-up period [25]. NLR and PLR can be easily calculated as the neutrophils-to-lymphocytes ratio and platelets-to-lymphocytes ratio in the peripheral blood and practical, inexpensive, and also valuable as high-cost inflammatory markers including IL-6, IL-1b, IL-8, and TNF-alpha.

In our data, 348 patients with ISSNHL had significantly different values of NLR and PLR from the control group of 537 healthy people which had been well matched (both *P* < 0.001). Other values like WBC, neutrophil, lymphocyte, monocyte, and glucose values were also seen significantly in the patients group, comparing with the control group (all *P* < 0.05). But neutrophil, lymphocyte, and monocyte values could be increased according to increased WBC value, which was still under acceptable normal value and could not be judged for an abnormal finding in the hematologic examination in both groups. Although glucose between the patients group and the control group was significantly different, because we could not control the sugar test with fasting glucose test for all patients and control group, we cannot be sure the value as the important risk factor. On the other hand, NLR and PLR values are more reliable risk factors because both values were increased in the patients group despite of increased lymphocyte counts. Some studies suggested the optimal cut-off value for NLR and PLR; NLR >3.95 and PLR >150 [8]. Our values of NLR and PLR between the patients group and the control group satisfied these cut-off criteria. The NLR values between the patients group and the control group were 4.48 ± 3.92 and 1.83 ± 0.87, and the PLR values were 169.25 ± 102.88 and 146.75 ± 55.14. This result may support a hypothesis that systemic stress activates inflammation, endothelial function is injured, impaired endothelium makes atherosclerosis represented by increasing NLR and PLR, and ischemic changes result in ISSNHL. It is true that the NLR and PLR can be related profoundly to ISSNHL.

To know prognostic factors for ISSNHL, we divided the patients group according to the recovery into subgroups: the recovered group and the unrecovered group. According to the previous study by Masuda et al., neutrophil and inflammatory markers levels were found higher in ISSNHL patients with no recovery [3]. Ulu et al. also showed that NLR levels were higher in patients without recovery [26]. In our study, comparing the recovered group with the unrecovered group, lymphocyte, monocyte, NLR, PLR, and glucose values were increased significantly for the unrecovered group (all *P* < 0.05). We further calculated odds ratios (OR) with 95% confidence intervals (CI) for unrecovered ISSNHL-risk factors with a binary logistic regression model. After adjustment for those values, only NLR value was associated strongly with the recovery of ISSNHL and was an independent risk factor for the improvement of PTAs later (*P* = 0.001). This result may show that the NLR value is more reflective of a prognosis of ISSNHL than the PLR value. PLR value could affect occurrences of ISSNHL but could not be related to the prognosis of ISSNHL. In the unrecovered group, the hearing losses at the frequencies (2000, 4000, and 8000 Hz) of the initial PTAs were occurred significantly, comparing to the recovery group (each *P* = 0.038, *P* < 0.001, and *P* < 0.001). There were hearing gains at all frequencies of the follow-up PTAs significantly in the recovered group that had the higher NLR value. But the recoveries were not big only at high frequencies (4000 and 8000 Hz) because the recovered patients had still difficulty for hearing at the high frequencies. The hearings at high frequencies may be more friable by vascular ischemic changes and be more difficult to be restored from the prior damage, while that at low and middle frequencies may be recovered well. If ISSNHL patients have a low NLR value, they may have a low tone loss tendency of PTA and be easy to be recovered.

A major limit of our study is the retrospective study that was considered. While a single baseline admission complete blood count (CBC) sampling has the benefit of being readily available, serial sampling may potentially yield a better analytical time point. For these reasons, new advanced, controlled, and randomized trials should be performed to establish and confirm our results. Nevertheless, with the large homogenous control group (*n* = 537) and well designed statistical data, we have suggested that NLR and PLR values help clinicians as practical and reliable indicator for ISSNHL patients in terms of the treatment and the predicting of the prognosis.

## 5. Conclusions

In the present study, although assigning specific mechanisms for the hearing loss of the ISSNHL patients are only speculative, we demonstrated for the first time that NLR and PLR values which are related to atherosclerosis in ISSNHL patients were significantly high and that endothelial dysfunction might play an important role in the physiopathology of hearing loss by the means of microvascular disturbances and inflammatory processes. Also the NLR level might be taken into account as a novel potential marker to predict the patients' prognosis in terms of recovery. Further prospective studies in this area will help to diagnosis more quickly and develop new treatments for ISSNHL patients.

## Figures and Tables

**Table 1:** Siegel's criteria of hearing improvement.

Type		Hearing recovery
I	Complete recovery	Patients whose final hearing level is better than 25 dB regardless of the size of the gain
II	Partial recovery	Patients who show more than 15 dB of gain and whose final hearing level is between 25 and 45 dB
III	Slight recovery	Patients who show more than 15 dB of gain and whose final hearing level is poorer than 45 dB
IV	No improvement	Patients who show less than 15 dB of gain

**Table 2:** The demographic and clinical characteristics of the study populations (patients and controls).

Variables	Patients group (*n* = 348)	Control group (*n* = 537)	*P* value
Age (yr)	48.19 ± 15.22	48.22 ± 11.60	0.976
Sex (*n*)			
Male	171	288	0.215
Female	177	249	
BMI (kg/m^2^)	23.47 ± 3.09	23.33 ± 4.45	0.804
Hb (10^3^/u)	14.50 ± 5.69	14.04 ± 1.45	0.074
WBC (10^3^/u)	8.42 ± 2.93	5.84 ± 1.47	**<0.001**
Neutrophil (10^3^/u)	7.15 ± 9.07	3.06 ± 1.25	**<0.001**
Lymphocyte (10^3^/u)	2.27 ± 3.31	1.77 ± 0.53	**0.005**
Monocyte (10^3^/u)	0.49 ± 0.76	0.31 ± 0.11	**<0.001**
NLR	4.48 ± 3.92	1.83 ± 0.87	**<0.001**
Platelet (10^3^/u)	252.40 ± 60.07	238.64 ± 49.98	**<0.001**
PLR	169.25 ± 102.88	146.75 ± 55.14	**<0.001**
Glucose (mg/dL)	111.71 ± 36.75	97.73 ± 19.81	**<0.001**
BUN (mg/dL)	14.55 ± 4.50	14.71 ± 5.30	0.647
Cr (mg/dL)	1.11 ± 5.48	0.82 ± 0.23	0.229
AST (U/L)	20.45 ± 13.37	21.77 ± 7.68	0.062
ALT (U/L)	22.10 ± 17.41	22.26 ± 14.83	0.888

The data are given as the means ± standard deviation or numbers and percentages; *P* < 0.05 value was accepted as significant level and the significant differences between the groups were shown in bold.

**Table 3:** The demographic and clinical characteristics of the recovered group and unrecovered group.

Variables	Recovered group (*n* = 210)	Unrecovered group (*n* = 138)	*P* value
Age (yr)	47.31 ± 15.34	49.53 ± 14.99	0.185
Sex (*n*)			
Male	97	0.189	0.189
Female	113	74	
BMI (kg/m^2^)	23.41 ± 2.36	23.56 ± 2.60	0.567
Hb (10^3^/u)	14.07 ± 1.71	15.17 ± 8.77	0.076
WBC (10^3^/u)	8.00 ± 2.86	10.82 ± 20.97	0.055
Neutrophil (10^3^/u)	6.47 ± 8.31	8.19 ± 10.07	0.083
Lymphocyte (10^3^/u)	2.62 ± 4.03	1.74 ± 1.57	**0.014**
Monocyte (10^3^/u)	0.56 ± 0.95	0.39 ± 0.25	0.067
NLR	3.50 ± 3.38	5.98 ± 4.22	**<0.001**
Platelet (10^3^/u)	252.74 ± 60.00	251.88 ± 60.38	0.896
PLR	148.47 ± 90.77	200.88 ± 112.12	**<0.001**
Glucose (mg/dL)	106.34 ± 27.28	119.87 ± 46.63	**0.002**
BUN (mg/dL)	14.25 ± 4.48	15.01 ± 4.50	0.122
Cr (mg/dL)	1.31 ± 7.05	0.81 ± 0.20	0.410
AST (U/L)	21.26 ± 15.51	19.22 ± 9.14	0.164
ALT (U/L)	23.21 ± 19.31	20.42 ± 13.95	0.119

The data are given as the means ± standard deviation or numbers and percentages; *P* < 0.05 value was accepted as significant level and the significant differences between the groups were shown in bold.

**Table 4:** Adjusted risk factors between the recovered group and the unrecovered group.

Variables	Adjusted odds ratio (95% CI)	*P* value
Lymphocyte (10^3^/u)	1.012 (0.856–1.197)	0.887
Monocyte (10^3^/u)	1.377 (0.558–3.400)	0.487
NLR	1.179 (1.069–1.299)	**0.001**
PLR	1.001 (0.997–1.004)	0.725
Glucose (mg/dL)	0.994 (0.987–1.000)	0.065

The factors were adjusted in a binary logistic regression model.

**Table 5:** Audiometric evaluation of the recovered group and the unrecovered group at different frequencies.

	Recovered group (*n* = 210)	Unrecovered group (*n* = 138)	*P* value
Follow-up periods (days)	16.26 ± 9.88	25.42 ± 12.54	**<0.001**
Initial PTA			
250 (Hz)	51.88 ± 28.68	49.78 ± 34.57	0.555
500 (Hz)	58.26 ± 26.46	56.27 ± 32.94	0.552
1000 (Hz)	58.17 ± 29.98	59.64 ± 32.88	0.667
2000 (Hz)	51.36 ± 32.87	58.95 ± 33.64	**0.038**
4000 (Hz)	51.50 ± 33.68	66.41 ± 33.42	**<0.001**
8000 (Hz)	56.05 ± 34.55	79.35 ± 32.18	**<0.001**
Follow-up PTA			
250 (Hz)	15.55 ± 14.93	36.97 ± 32.09	**<0.001**
500 (Hz)	17.83 ± 19.01	43.95 ± 31.26	**<0.001**
1000 (Hz)	20.50 ± 22.09	45.88 ± 31.25	**<0.001**
2000 (Hz)	20.93 ± 23.77	45.51 ± 31.01	**<0.001**
4000 (Hz)	28.55 ± 28.06	54.93 ± 30.17	**<0.001**
8000 (Hz)	38.89 ± 32.36	71.73 ± 29.85	**<0.001**

The data are given as the means ± standard deviation or numbers and percentages; *P* < 0.05 value was accepted as significant level and the significant differences between the groups were shown in bold. PTA: pure tone average.

## References

[1] Hughes GB, Freedman MA, Haberkamp TJ, Guay ME (1996). Sudden sensorineural hearing loss. *Otolaryngologic Clinics of North America*.

[2] Merchant SN, Durand ML, Adams JC (2008). Sudden deafness: is it viral?. *Journal of Oto-Rhino-Laryngology and Its Related Specialties*.

[3] Masuda M, Kanzaki S, Minami S (2012). Correlations of inflammatory biomarkers with the onset and prognosis of idiopathic sudden sensorineural hearing loss. *Otology & Neurotology*.

[4] Ryan AF, Harris JP, Keithley EM (2002). Immune-mediated hearing loss: basic mechanisms and options for therapy. *Acta Oto-Laryngologica*.

[5] Hiramatsu M, Teranishi M, Uchida Y (2012). Polymorphisms in genes involved in inflammatory pathways in patients with sudden sensorineural hearing loss. *Journal of Neurogenetics*.

[6] Hoffman M, Blum A, Baruch R, Kaplan E, Benjamin M (2004). Leukocytes and coronary heart disease. *Atherosclerosis*.

[7] Papa A, Emdin M, Passino C, Michelassi C, Battaglia D, Cocci F (2008). Predictive value of elevated neutrophil-lymphocyte ratio on cardiac mortality in patients with stable coronary artery disease. *Clinica Chimica Acta*.

[8] Gary T, Pichler M, Belaj K (2013). Platelet-to-lymphocyte ratio: a novel marker for critical limb ischemia in peripheral arterial occlusive disease patients. *PLoS ONE*.

[9] Kwon H, Kim SH, Oh SY (2012). Clinical significance of preoperative neutrophil-lymphocyte versus platelet-lymphocyte ratio in patients with operable colorectal cancer. *Biomarkers*.

[10] Stachler RJ, Chandrasekhar SS, Archer SM (2012). Clinical practice guideline: sudden hearing loss. *Otolaryngology-Head and Neck Surgery*.

[11] Siegel LG (1975). The treatment of idiopathic sudden sensorineural hearing loss. *Otolaryngologic Clinics of North America*.

[12] Wilson WR, Byl FM, Laird N (1980). The efficacy of steroids in the treatment of idiopathic sudden hearing loss. A double-blind clinical study. *Archives of Otolaryngology*.

[13] Toubi E, Halas K, Ben-David J, Sabo E, Kessel A, Luntz M (2004). Immune-mediated disorders associated with idiopathic sudden sensorineural hearing loss. *Annals of Otology, Rhinology and Laryngology*.

[14] Weng SF, Chen YS, Liu TC, Hsu CJ, Tseng FY (2004). Prognostic factors of sudden sensorineural hearing loss in diabetic patients. *Diabetes Care*.

[15] Rudack C, Langer C, Stoll W, Rust S, Walter M (2006). Vascular risk factors in sudden hearing loss. *Thrombosis and Haemostasis*.

[16] Mosnier I, Stepanian A, Baron G (2010). Cardiovascular and thromboembolic risk factors in idiopathic sudden sensorineural hearing loss: a case-control study. *Audiology and Neurotology*.

[17] Chau JK, Lin JRJ, Atashband S, Irvine RA, Westerberg BD (2010). Systematic review of the evidence for the etiology of adult sudden sensorineural hearing loss. *Laryngoscope*.

[18] Ciccone MM, Cortese F, Pinto M (2012). Endothelial function and cardiovascular risk in patients with idiopathic sudden sensorineural hearing loss. *Atherosclerosis*.

[19] Capaccio P, Cuccarini V, Ottaviani F, Fracchiolla NS, Bossi A, Pignataro L (2009). Prothrombotic gene mutations in patients with sudden sensorineural hearing loss and cardiovascular thrombotic disease. *Annals of Otology, Rhinology and Laryngology*.

[20] Tamhane UU, Aneja S, Montgomery D, Rogers E, Eagle KA, Gurm HS (2008). Association between admission neutrophil to lymphocyte ratio and outcomes in patients with acute coronary syndrome. *American Journal of Cardiology*.

[21] Kuyumcu ME, Yesil Y, Oztürk ZA (2012). The evaluation of neutrophil-lymphocyte ratio in Alzheimer’s disease. *Dementia and Geriatric Cognitive Disorders*.

[22] Chen T-M, Lin C-C, Huang P-T, Wen C-F (2012). Neutrophil-to-lymphocyte ratio associated with mortality in early hepatocellular carcinoma patients after radiofrequency ablation. *Journal of Gastroenterology and Hepatology*.

[23] Imtiaz F, Shafique K, Mirza S, Ayoob Z, Vart P, Rao S (2012). Neutrophil lymphocyte ratio as a measure of systemic inflammation in prevalent chronic diseases in Asian population. *International Archives of Medicine*.

[24] Ott I, Neumann F-J, Gawaz M, Schmitt M, Schömig A (1996). Increased neutrophil-platelet adhesion in patients with unstable angina. *Circulation*.

[25] Tasoglu I, Sert D, Colak N, Uzun A, Songur M, Ecevit A (2013). Neutrophil-lymphocyte ratio and the platelet-lymphocyte ratio predict the limb survival in critical limb ischemia. *Clinical and Applied Thrombosis/Hemostasis*.

[26] Ulu S, Ulu MS, Bucak A, Ahsen A, Yucedag F, Aycicek A (2013). Neutrophil-to-lymphocyte ratio as a new, quick, and reliable indicator for predicting diagnosis and prognosis of idiopathic sudden sensorineural hearing loss. *Otology & Neurotology*.

